# Triazole-Modified Peptidomimetics: An Opportunity for Drug Discovery and Development

**DOI:** 10.3389/fchem.2021.674705

**Published:** 2021-05-20

**Authors:** Agnieszka Staśkiewicz, Patrycja Ledwoń, Paolo Rovero, Anna Maria Papini, Rafal Latajka

**Affiliations:** ^1^Department of Bioorganic Chemistry, Faculty of Chemistry, Wroclaw University of Science and Technology, Wroclaw, Poland; ^2^Interdepartmental Research Unit of Peptide and Protein Chemistry and Biology, Department of Chemistry “Ugo Schiff”, University of Florence, Firenze, Italy; ^3^Interdepartmental Research Unit of Peptide and Protein Chemistry and Biology, Department of Neurosciences, Psychology, Drug Research and Child Health-Section of Pharmaceutical Sciences and Nutraceutics, University of Florence, Firenze, Italy

**Keywords:** 1,2,3-triazole, 1,2,4-triazole, CuAAC, antibacterial triazoles, antifungal triazoles, antiviral triazoles, disulphide bond mimetic, enzyme inhibitors

## Abstract

Peptidomimetics play a fundamental role in drug design due to their preferential properties regarding natural peptides. In particular, compounds possessing nitrogen-containing heterocycles have been intensively studied in recent years. The triazolyl moiety incorporation decreases the molecule susceptibility to enzymatic degradation, reduction, hydrolysis, and oxidation. In fact, peptides containing triazole rings are a typical example of peptidomimetics. They have all the advantages over classic peptides. Both efficient synthetic methods and biological activity make these systems an interesting and promising object of research. Peptide triazole derivatives display a diversity of biological properties and can be obtained via numerous synthetic strategies. In this review, we have highlighted the importance of the triazole-modified peptidomimetics in the field of drug design. We present an overview on new achievements in triazolyl-containing peptidomimetics synthesis and their biological activity as inhibitors of enzymes or against cancer, viruses, bacteria, or fungi. The relevance of above-mentioned compounds was confirmed by their comparison with unmodified peptides.

## Introduction

Peptide-based treatments play a fundamental role in the drug market as a matter of their plentiful properties. Peptides are a group of compounds possessing limited immunogenic activity and due to the small size are able to penetrate tissues and organs. Moreover, their synthesis is relatively easy and inexpensive. However, peptides exhibit reduced metabolic stability and bioavailability *in vivo* (Baharloui et al., 2019). Therefore, the development of chemically modified peptides, generically defined peptidomimetics, gained increasing importance in recent years (Sun et al., 2015). Peptidomimetics are molecules able to mimic natural peptides and proteins. Structures of peptidomimetics can preserve the capability for interactions with the biological targets and display identical *in vivo* effects of the corresponding unmodified peptides (Mabonga and Kappo, 2020).

Nowadays, pharmaceutical companies are focused on research and development of novel and harmless drugs into the market. Thus, researchers are stimulated to design novel structures, making an effort to redeem these issues. The chemistry of peptides and heterocycles is challenging in this field. In particular, nitrogen-containing heterocycles became popular in the past years (Sun et al., 2015) and therefore, they are present in numerous drug molecules.

**Figure 1:**
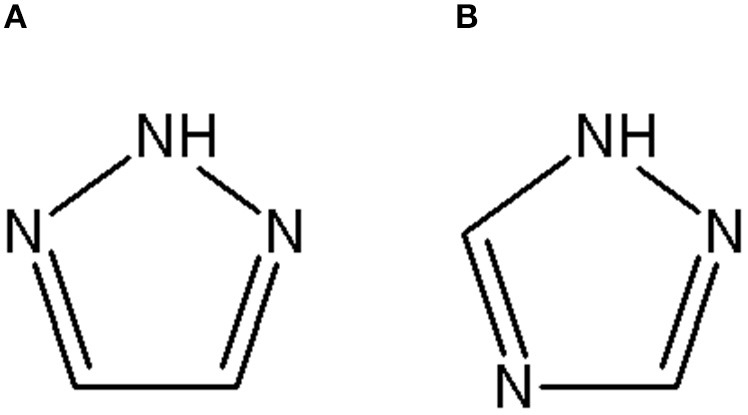
Structures of the two isomeric forms of triazoles. **(A)** 1,2,3-triazole; **(B)** 1,2,4-triazole (Souza and Miranda, 2019).

Triazoles are five-membered aromatic heterocyclic moieties, which possess three nitrogen and two carbon atoms in the structure (Costa et al., 2017). They occur in two tautomeric forms: the 1,2,3-triazole or the 1,2,4-triazole (Figure 1) depending on the position of the NH group in the ring (Souza and Miranda, 2019).

It was previously reported that compounds including triazolyl moieties in the structure display interesting properties in medicine, pharmacology, and medicinal chemistry. Triazoles are structures stable to hydrolysis, oxidation, and reduction conditions. They attracted the attention of researchers because of their low toxicity and relatively easy synthesis in high yields (Costa et al., 2017). The 1,4-disubstitued 1,2,3-triazolyl core structure is mimetic of *trans-*amide bond regarding to planarity, dipole moment, comparable size, and capability to hydrogen bond formation (Valverde et al., 2013). According to the literature, the triazole derivatives retain a variety of biological functions. They can act against cancer, microorganisms, including bacteria, fungi, and viruses, e.g., human immunodeficiency virus (HIV).

In this review, we will focus on the triazole-modified peptidomimetics, which were investigated as potential drugs and therapeutics in the past.

## Chemical Background

### The Triazole Ring Mimicking the Disulphide Bond in Cyclic Peptides

The disulphide bond plays a relevant role in the folding process of peptides and proteins. The S-S bridge can stabilise and rigidify the 3D structure through the macrocycle formation (Holland-Nell and Meldal, 2011; Testa et al., 2018a). Moreover, disulphide bonds can play a relevant role in the biological activity, enhancing selectivity and metabolic stability of proteins (Liu et al., 2019). Protein conformation is sometimes constrained by one or multiple S-S bonds, which improve enzymatic and chemical stability. Nevertheless, the disulphide bridge is prone to redox reactions in the presence of thiol-containing compounds, such as oxidoreductases, serum albumin, and glutathione. The loss of conformational constraint can lead to metabolic degradation, peptide inactivation, and decreased drug efficacy (White et al., 2020). Accordingly, several methods have been examined in order to replace the disulphide bridge with metabolically stable surrogates, for instance, diselenide, tioether, hydrocarbon-based linkers, and triazoles (Cui et al., 2013). Triazoles display chemical orthogonality and ensure high stability against proteases and isomerases. Moreover, the triazolyl moieties can be obtained via two-component reaction, which is comparable to forming the disulphide bond starting from two cysteine residues. The triazole can be generated from an alkyne and an azide by copper-catalysed Huisgen cycloaddition (CuAAC) (Holland-Nell and Meldal, 2011).

Based on these concepts, CuAAC strategy has been also used to form triazolyl side chain-to-side chain bridge(s) to introduce conformational constraints in linear peptides, aimed to stabilise their bioactive conformation. The first attempt to stabilise the α-helical conformation of a model peptide by side chain-to-side chain cyclisation, CuAAC was applied by Cantel et al. to a parathyroid hormone-related peptide (PTHrP) analogue (Cantel et al., 2008). Several subsequent studies from the same group (Scrima et al., 2010) and other laboratories, followed similar approach to different bioactive peptides, as recently reviewed (Testa et al., 2018b).

### Preparation and Synthesis of Triazole-Modified Peptides Including the Triazolyl Core

The substituted 1,2,4-triazoles and their analogues are common structural modifications in many organic and bioactive molecules. According to the literature, diverse approaches have been developed for the synthesis of the triazole motif (Xu et al., 2015).

**Figure 2:**
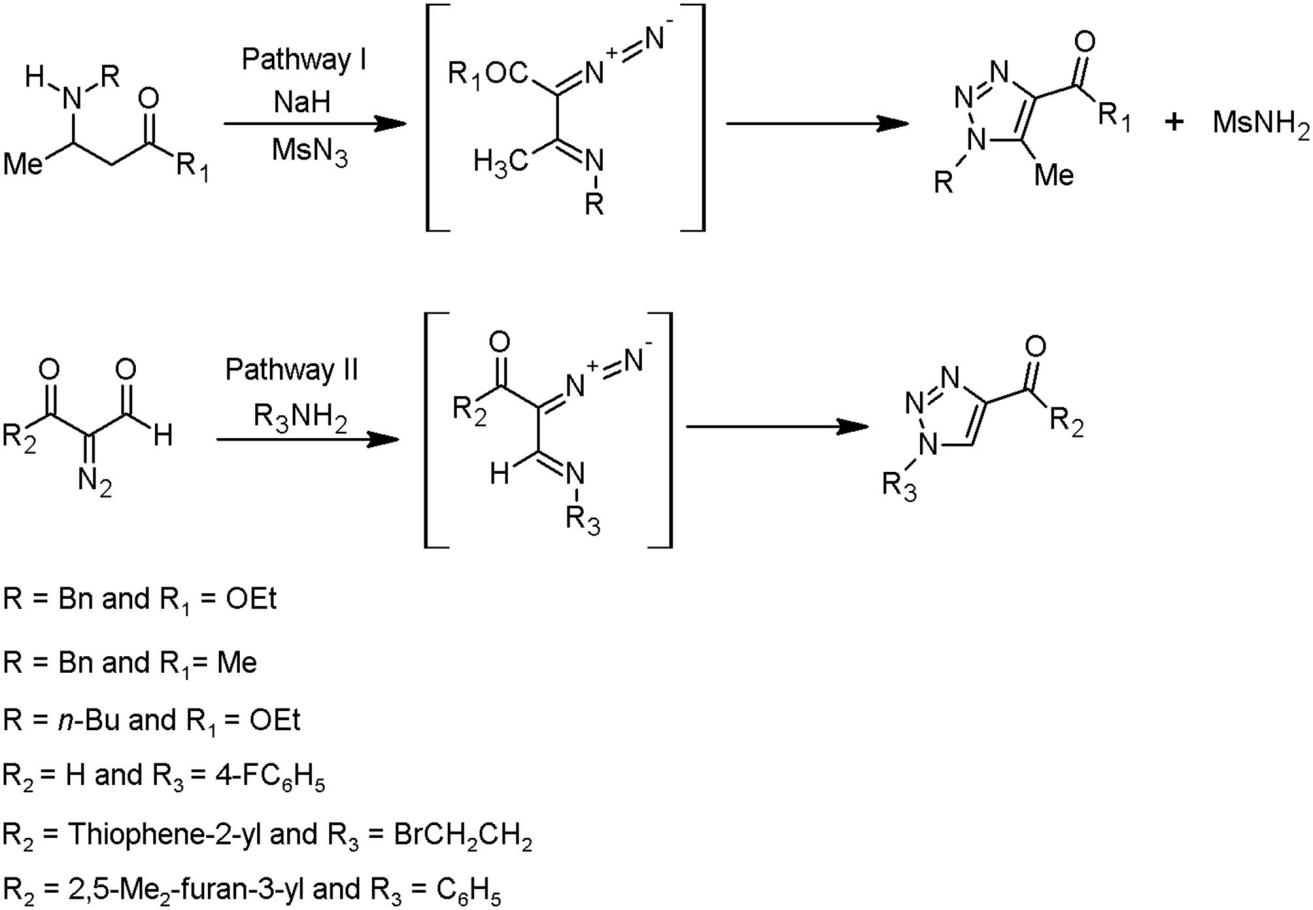
Methods to prepare the triazoles from α-diazoimines (Jordão et al., 2011).

Overall, it is known that two synthetic techniques can be applied in the framework of the triazole moieties design. One of them is Huisgen cycloaddition catalysed by copper(I) ions, as further described in this chapter. This method is characterised by an intramolecular 1,5-electrocyclisation of β-substituted α-diazocarbonyl compounds. The conditions of subsequent process are less restricted and require mainly commercially available starting materials. Based on this methodology, two pathways have been characterised by Jordão et al. (2011), starting from: (i) β-amino-α,β-unsaturated ketones or esters, followed by a diazo transfer reaction (pathway **I**) and (ii) α-diazocarbonyl compounds, followed by α-diazoimino formation (pathway **II**) (Figure 2).

The diazo-donor reagents can be sulfonyl azides, for instance, *p*-carboxylbenzenesulfonyl azide, methanesulfonyl azide, tosyl azide, and 3-diazo-1,3-dihydro-2*H-*indol-2-one (Jordão et al., 2011).

Nagasawa et al. have developed the first method for the transition metal catalysed preparation of 1,2,4-triazoles. They used the effective copper-catalysed synthesis of the 1,2,4-triazole analogues by coupling nitriles with amidines (Ueda and Nagasawa, 2009). Moreover, the research group of Beifuss discovered the efficient and novel copper-catalysed cascade reaction between ammonium carbonate and imidates for the preparation of symmetrically substituted 3,5-diaryl-1,2,4-triazoles (Sudheendran et al., 2014). The procedures presented above were effective but most of them considered the substrates, such as imidates or amidines synthesised from nitriles (Creary and Sky, 1990). Zhang et al. have described the catalytic transformation of alkynes and azides mediated by ruthenium(II) ion (RuAAC), which gives selective admission to 1,5-disubstitued 1,2,3-triazoles as a structural variation (Zhang et al., 2005).

The CuAAC reaction was the first to be carried out to create the five-membered heterocycle compounds, including the triazole moiety, without the use of complex reagents. The azide-alkyne cycloaddition was rapidly recognised as the most effective strategy to conjugate two or more structural motifs (Ganesh et al., 2011).

**Figure 3:**
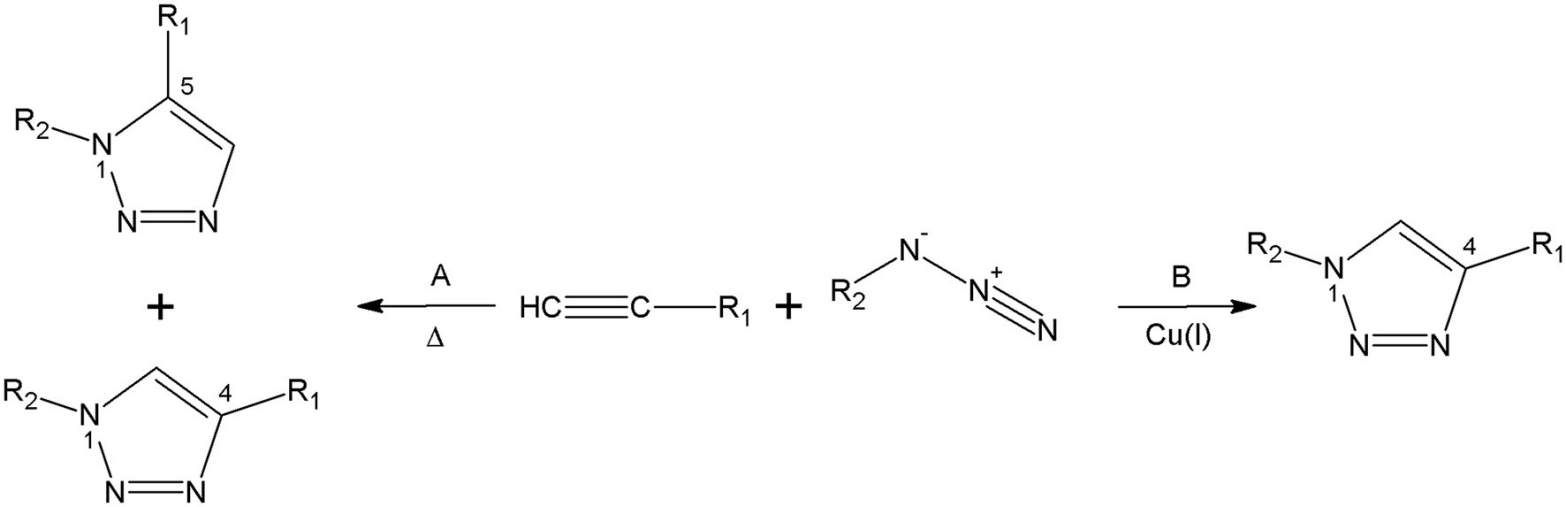
The Huisgen cycloaddition. **(A)** Under thermal conditions and **(B)** catalysed by Cu(I) ions.

The foremost method for the triazolyl core synthesis was described by Huisgen (1963). The cycloaddition of alkynes and azides is an extremely atom-economical reaction, leading to the formation of the 1,2,3-triazoles. Nevertheless, the thermal process is not regioselective and the result is the formation of both 1,4 and 1,5-disubstituted 1,2,3-triazoles (Akula and Lakshman, 2012). The reaction was performed between an acetylene and the azide under reflux conditions in toluene, leading to a mixture of 1,4 and 1,5-regioisomers of 1,2,3-triazoles (Souza and Miranda, 2019). In 2002, Meldal and Sharpless and their colleagues independently reported the most extensively used click chemistry method (Rostovtsev et al., 2002; Tornøe et al., 2002). The cycloaddition catalysed by copper(I) ions leads to 1,4-disubstitued 1,2,3-triazoles under undemanding conditions (Figure 3; Struthers et al., 2010).

The copper-catalysed azide-alkyne cycloaddition is efficient, selective, and is performed in a mild reaction environment. This method may be conducted both in organic solvents and in water with almost comprehensive conversion and selectivity (Struthers et al., 2010). The cycloaddition requires Cu(I) or Cu(II) salts in a mixture of *tert*-butyl alcohol and water or organic solvents at room temperature (Totobenazara and Burke, 2015). The above-mentioned technique is one of the examples of click reaction proceeding with high yield and purity, regioselectivity, and comprehensive chemical conversion (Kaushik et al., 2015). The 1,2,3-triazole is well-known as a versatile peptidomimetic moiety (Valverde et al., 2012).

Kuang et al. have designed the reaction in non-reductive conditions, involving N-containing additional ligands and Cu(OAc)_2_. Then the azide chelation enhances meaningfully the reaction rate (Kuang et al., 2010).

Homogeneous silver(I) catalysed azide-alkyne cycloaddition (AgAAC) was studied by McNulty et al. due to the toxicity of redox-active copper(I) ions for many biological functions. The reaction between a thermally stable phosphane ligand and silver acetate was carried out at room temperature. However, heating up to 90°C was demonstrated to increase the yield (McNulty and Keskar, 2012).

Smith et al. reported a zinc mediated azide-alkyne cycloaddition forming 1,5 and 1,4,5-substitued 1,2,3-triazoles at room temperature. The reaction was conducted with a catalytic amount of N-methylimidazole, which is obligatory to create the zinc acetylide (Smith and Greaney, 2013).

The research group of Fokin has reported the first transition metal-free catalytic azide-alkyne cycloaddition for the synthesis of 1,5-diaryl-1,2,3-triazoles. The triazolyl moiety was formed between terminal alkynes and aryl azides at room temperature in the presence of a catalytic amount of hydroxide, in high yield. This reaction is easy and insensitive to moisture or atmospheric oxygen (Kwok et al., 2010).

Moreover, the techniques involving ultrasounds have the benefit of allowing to perform the reactions in both homogeneous and heterogeneous systems (Totobenazara and Burke, 2015). Cintas et al. presented the synthesis of 1,4-disubstitued 1,2,3-triazole analogues via metallic copper under ultrasounds without the addition of any ligand (Cintas et al., 2010).

The triazole core is considered as a common peptidomimetic moiety, able to accommodate any peptide secondary structure. The synthesis of triazole amide surrogates by a peptidomimetic ligation approach requires N- and C-terminally modified peptides with α-azido acids and α-amino alkynes, respectively. The synthesis of chiral α-amino alkynes can be performed in a few steps starting from the protected parent amino acids. The incorporation of 1,4-disubtitued triazoles into the backbone of a long peptide chain was achieved by classical solid-phase peptide synthetic strategies (SPPS), involving triazole-containing pseudo-dipeptides as building blocks (Valverde et al., 2012).

**Figure 4:**
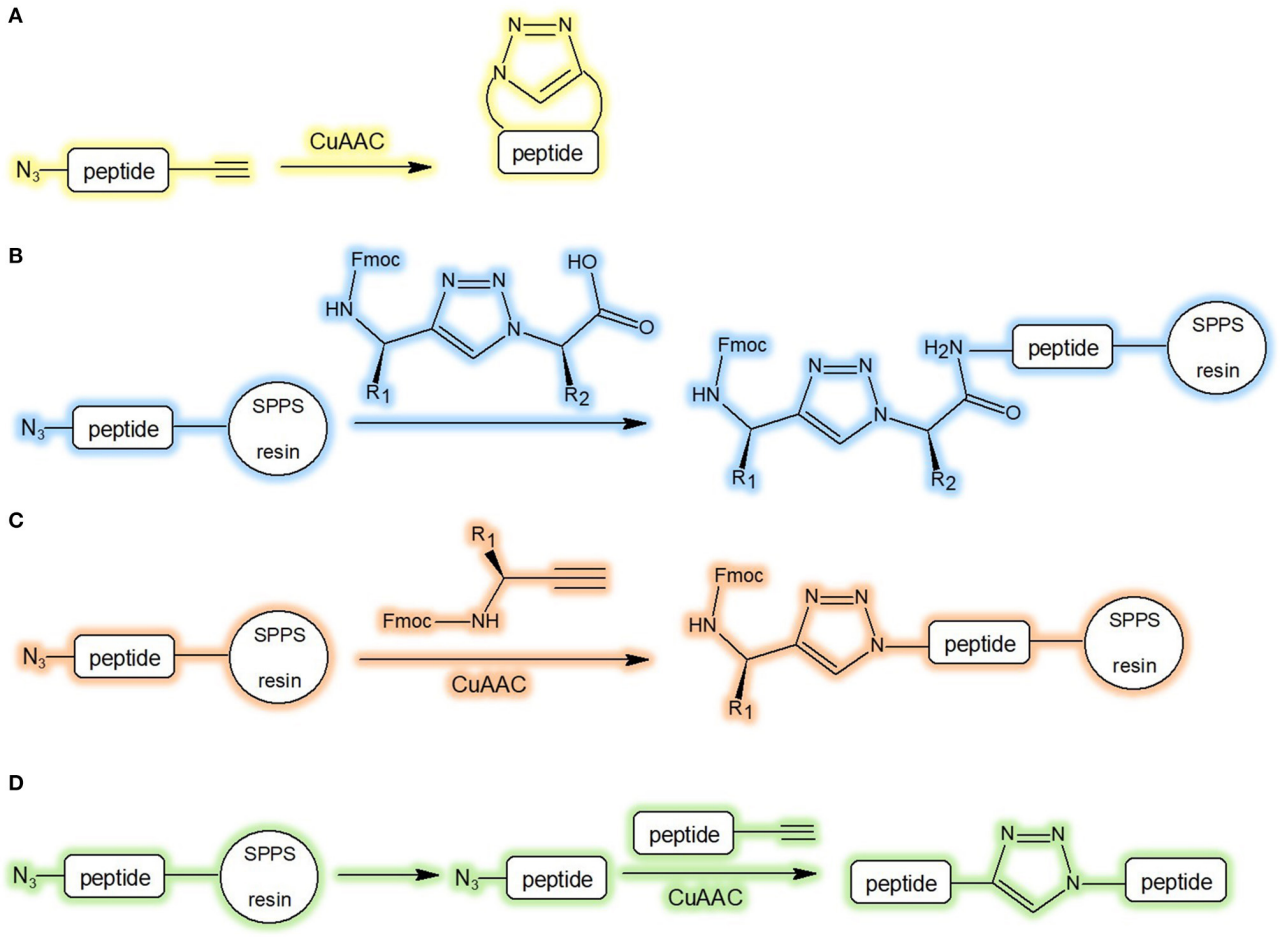
Synthetic strategies of triazolyl containing cyclopeptidomimetics. **(A)** CuAAC in solution-phase peptide synthesis; **(B)** incorporation of triazole during SPPS; **(C)** attachment of triazole to the solid support by CuAAC; **(D)** conjugation of peptide fragment after CuAAC in solution (Valverde and Mindt, 2012).

The preparation of low molecular weight triazolyl cyclopeptidomimetics is generally carried out by peptide synthesis in solution, via the precursor followed by CuAAC (Figure 4A). In case of longer sequences, the triazole moiety can be easily incorporated into the chain during peptide elongation by conventional SPPS coupling method (Figure 4B). Differently, the heterocycle structures can be attached on the solid support by the copper catalysed azide-alkyne cycloaddition of the azide with the alkyne precursor (Figure 4C). The peptide fragments after CuAAC, performed from alkyne and azide, can be conjugated in solution. This method is useful for large proteins as well (Valverde and Mindt, 2012; Figure 4D). Anyway, the synthesis of the building blocks N(alpha)-Fmoc-protected omega-azido and omega-ynoic-alpha-amino acids (Le Chevalier Isaad et al., 2008) was demonstrated to be instrumental to set up valuable synthetic protocols (Le Chevalier-Isaad et al., 2009) to develop a large variety of “Clickable” peptides (Testa et al., 2014).

Microwave-assisted click chemistry has also been studied. The microwave irradiation reduces the reaction time, enables effective internal heat transfer, and consequently the yield is increased. High temperature is reached rapidly, therefore polymerization and decomposition are prevented (Totobenazara and Burke, 2015). Ma et al. have reported a microwave-assisted copper-mediated one-pot three-component synthesis of symmetrically substituted 1,2,4-triazoles from aryl nitriles and amines. This synthetic method requires two equivalents of copper salts and the reaction yield was ~12-55% (Xu et al., 2015). D'Ercole et al. have developed an effective and reproducible microwave-assisted approach to the synthesis of side chain-to-side chain cyclopeptides. In the context of the synthesis of clicked H1-relaxin analogues containing the binding cassette, the azide-alkyne cycloaddition catalysed by copper(I) ions was performed in solid-phase. All the crucial parameters, for instance solvent, type of resin, catalytic system, reaction time, and microwave energy were optimised by a systemic one factor at a time method. This technique is a profitable tool to prepare libraries of conformationally constrained derivatives of relaxin peptide analogues (D'Ercole et al., 2020).

Moreover, 1,4-disubtitued 1,2,3-triazoles are similar to substituted imidazoles due to their coordinative properties, and also to amide bonds in terms of planarity and molecular dimension. The triazole motifs are prospectively universal ligands providing donor sites for diverse metal ions coordination (Struthers et al., 2010). Synthetic techniques to obtain molecular structures containing triazolyl moieties reported in this review are summarised in the Table 1.

**Table 1:** Synthetic techniques to obtain molecular structures containing triazolyl moieties reported in this review.

	**Method of synthesis**	**Yield [%]**	**References**
Metal catalysed reaction	CuAAC RuAAC AgAAC Zinc-catalysed azide-alkyne cycloaddition Copper-catalysed cascade reaction Transition metal catalysed reaction Ultrasound copper-catalysed reaction with no ligands	– – 68–99 49–76 64–68 52–85 ≈80	Huisgen, 1963 Zhang et al., 2005 McNulty and Keskar, 2012 Smith and Greaney, 2013 Sudheendran et al., 2014 Ueda and Nagasawa, 2009 Cintas et al., 2010
SPPS	Pseudo-dipeptide containing building blocks	–	Valverde et al., 2012
Microwave-assisted reaction	Copper-mediated one-pot three-component synthesis Side chain-to-side chain cyclisation	12–55 25.5	Xu et al., 2015 D'Ercole et al., 2020
Others	1,5-electrocyclisation of β-substituted α-diazocarbonyl compounds Non-reductive conditions Metal free azide-alkyne cycloaddition	47–93 76–99 37–92	Jordão et al., 2011 Kuang et al., 2010 Kwok et al., 2010

## The Triazoles as Agents Against Microorganisms

It is not surprising that the CuAAC has been applied in various fields of chemistry, for instance, organic synthesis and medicinal chemistry (Souza and Miranda, 2019). The structures containing the triazole motif have been extensively investigated for pharmaceutical applications. The 1,2,3-triazole analogues show various bioactive properties, such as antifungal or antibacterial ones (Nasli-Esfahani et al., 2019). These peptidomimetics with antimicrobial properties displayed host defence functions. They are able to interact with biological membranes and modulate the immune system (Junior et al., 2017).

Tachyplesin I (TPI-1), a β-hairpin antimicrobial peptide, is a 17-residues bicyclic peptide with high antimicrobial activity. The conformation of TPI is stabilised by two cross-strand disulphide bonds. The bioactivity of TPI-1 analogues was studied against gramme positive bacteria, such as *Bacillus subtilis, Staphylococcus epidermidis*, and three negative controls: *Salmonella enterica, Pseudomonas aeruginosa*, and *Escherichia coli*. Some bacterial strains were grown in the presence of high concentrations of the TPI-1 analogues (Cui et al., 2013). In the Table 2, are presented the structures of TPI derivatives including the triazolyl modifications and the results obtained in biological assays.

**Table 2:** The structures of TPI analogues with minimal inhibitory concentrations (MIC) values by the standard 2-fold dilution protocol in μg × mL^−1^ (Cui et al., 2013).

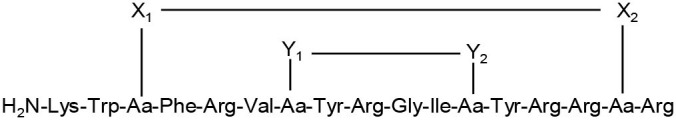									
**Compound**	**X**_**1**_	**X**_**2**_	**Y**_**1**_	**Y**_**2**_	***Bacillus subtilis***	***Staphylococcus epidermidis***	***Salmonella enterica***	***Pseudomonas aeruginosa***	***Escherichia coli***
TPI-1	S	S	S	S	4	4	8	16	8
TPI-2	CH_2_	CH_2_	CH_2_	CH_2_	8	32	64	256	16
TPI-3	S	CH_2_	S	CH_2_	16	16	64	128	16
TPI-4	CH_2_	CH_2_	S	S	8	16	32	64	16
TPI-5	S	S	CH_2_	CH_2_	8	16	64	64	16
TPI-6	S	CH_2_	S	S	8	8	64	64	64
TPI-7	S	S	S	CH_2_	8	4	32	16	8
TPI-8	S	S	CH_2_	S	16	16	32	64	32
TPI-9	CH_2_	S	S	S	4	4	16	16	8
TPI-triazole	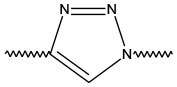		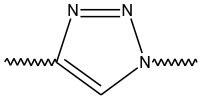		5.5	8	–	–	10
TPI-triazole'	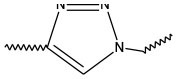		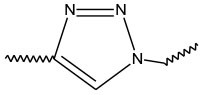		4.5	10.5	–	–	7

Despite the fact that the values obtained for TPI-2 and TPI-3 analogues were deviated from the ones for the ordered β-pleat sheet structures, it was observed that they were still efficient inhibitors of bacterial growth (MIC values 2 to 16-fold lower than for TPI-1). Moreover, it was reported that neither the incorrectly cyclised nor the linear analogues of TPI-1 showed significant antimicrobial activity (Cui et al., 2013).

### Antibacterial Properties of Triazole Peptide Derivatives

Among many bioactive properties, the 1,2,3-triazolyl derivatives can act as antibacterial agents. Liu et al. have used the click chemistry to convert the Polybia-MPI (MPI), a natural antimicrobial peptide (AMP) isolated from the venom of the social wasp *Polybia paulista*. It possesses weak haemolytic features and activity against gramme positive and negative bacterial strains. The authors have synthesised two intramolecular cyclic analogues containing the 1,2,3-triazolyl moiety, C-MPI-1 and C-MPI-2, with different bridge orientation with the goal of enhancing the MPI stability (Liu et al., 2017b). The study outcomes are presented in the Table 3.

**Table 3:** Amino acid sequence and antibacterial activities of MPI and its analogues with MIC values given in μM (NA, no antimicrobial activity) (Liu et al., 2017b).

**Peptide**	**Sequence**	***Escherichia coli***	***Staphylococcus aureus***	***Bacillus subtilis***	***Pseudomonas aeruginosa***
MPI	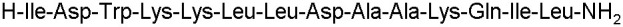	32	32	8	NA
C-MPI-1	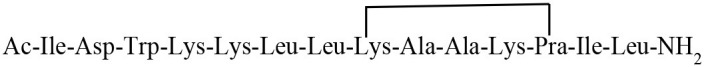	64	64	8	NA
C-MPI-2	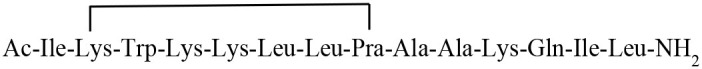	128	256	128	NA
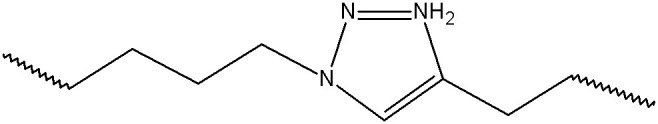					

The structure C-MPI-1, cyclised at the *i* to *i*+4 positions, presents an improved helical tendency in H_2_O, 50% trifluoroethyl alcohol (TFE), 30 mM sodium dodecyl sulphate (SDS), and enhanced stability against trypsin in comparison to MPI (parent peptide). On the other hand, the analogue C-MPI-2, cyclised at the *i* to *i*+6 positions, lost helical structure in the same environment. Therefore, the importance of the peptides helicity in antimicrobial activity was confirmed (Liu et al., 2017b).

Moreover, Lui et al. described an efficient click chemistry strategy to enhance *in vivo* and *in vitro* antimicrobial activity of several peptides. Anoplin and cross-linked sequences, such as anoplin analogue J-AA, the hexapeptide J-RR (RRWWRF), and their chimaera J-AR were examined. All the designed analogues remarkably increased the antimicrobial activity against bacteria strains, such as *E. coli, S. aureus, P. aeruginosa*, and *B. subtilis* (Table 4).

**Table 4:** The structures of anoplin and its analogues with antibacterial activities with MIC values given in μM (Liu et al., 2017a).

**Peptide**	**Sequence**	***Escherichia coli***	***Staphylococcus aureus***	***Pseudomonas aeruginosa***	***Bacillus subtilis***
Anoplin	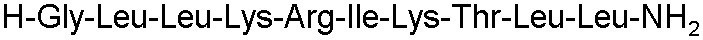	64	64	16	16
J-AA	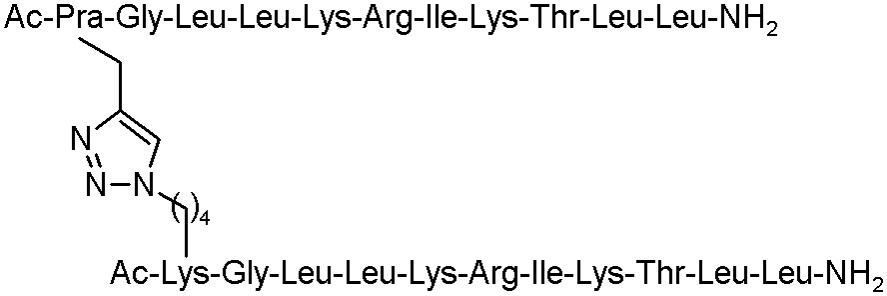	4	8	16	2
J-RR	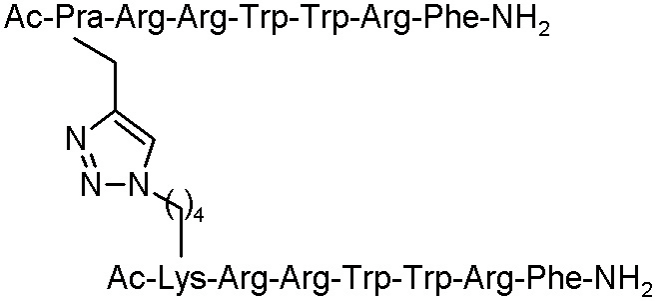	8	4	16	4
J-AR	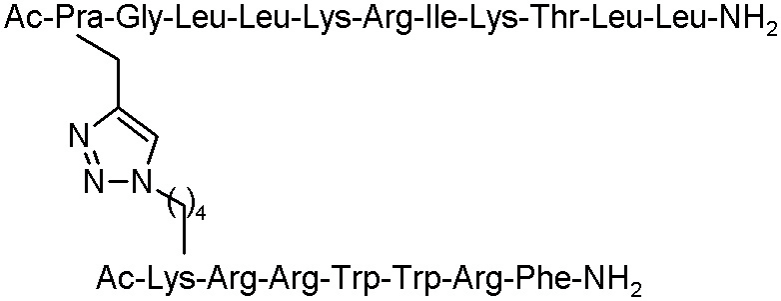	8	4	16	4

Furthermore, these peptides cross-linked by a triazole bridge, forming an α-helical structure in 50% TFE conditions, were able to kill the bacteria rapidly by membrane disruption. The toxicity study of all analogues presented that J-AR and J-RR did not display any toxicity in case of mature mice exposed to concentration up to 120 mg/Kg and the 50% lethal dose (LD_50_) of J-AA (53.6 mg/Kg). Additionally, the mice treated with these peptidomimetics had lower degree of bacterial load than the control group in the mouse model infected by *Escherichia coli*. The structure J-RR exhibited high efficiency in decreasing blood bacterial counts infected by *Methicillin-resistant Staphylococcus aureus* (MRSA) strain in the mouse model. These results can be the starting point for the development of drug candidates in the future (Liu et al., 2017a).

### Antifungal Activity of Peptides Including 1,2,3-Triazolyl Moieties in the Structures

Pathological states caused by pathogenic fungi are among the most serious diseases (Zhang et al., 2016). In the last years, fungal infections became a severe medical task because of many clinical challenges, such as transplantation, HIV infections, application of immunosuppressive agents, and cancer (Sadeghpour et al., 2017). Structures containing the 1,2,3-triazolyl core play an important function due to the wide therapeutic applications in antifungal therapy. For this reason, the triazole-containing drugs have been effectively developed and applied in the treatment of numerous microbial infections for years (Yu et al., 2015). An expanding resistance to the present antifungal treatments led to the development of novel triazole derivatives with advanced therapeutic indexes and enhanced antifungal spectrum (Wang et al., 2014).

The azoles possess an activity against fungi by inhibition of cytochrome P450 14α-demethylase (CYP51). This enzyme is relevant in the pathway of ergosterol biosynthesis from lanosterol in fungi and yeasts (Sadeghpour et al., 2017). The CYP51 contains an iron protoporphyrin unit situated in the active site. It catalyses the oxidative removal of the 14α-methyl group of lanosterol through characteristic activity of monooxygenase. The azole nitrogen compounds as antifungal agents can block the fungal ergosterol biosynthesis by binding to the iron ion of the porphyrin. This action is performed by preventing the penetration of the lanosterol to the active site of the enzyme. The decrease of the 14α-methylated sterols aggregation with the ergosterol adjusts the fluency of membranes, reduction of extended penetrability, and activity of membrane-associated enzymes. This phenomenon leads to the inhibition of fungal cells growth (Sadeghpour et al., 2017) and replication (Yu et al., 2014).

**Figure 5:**
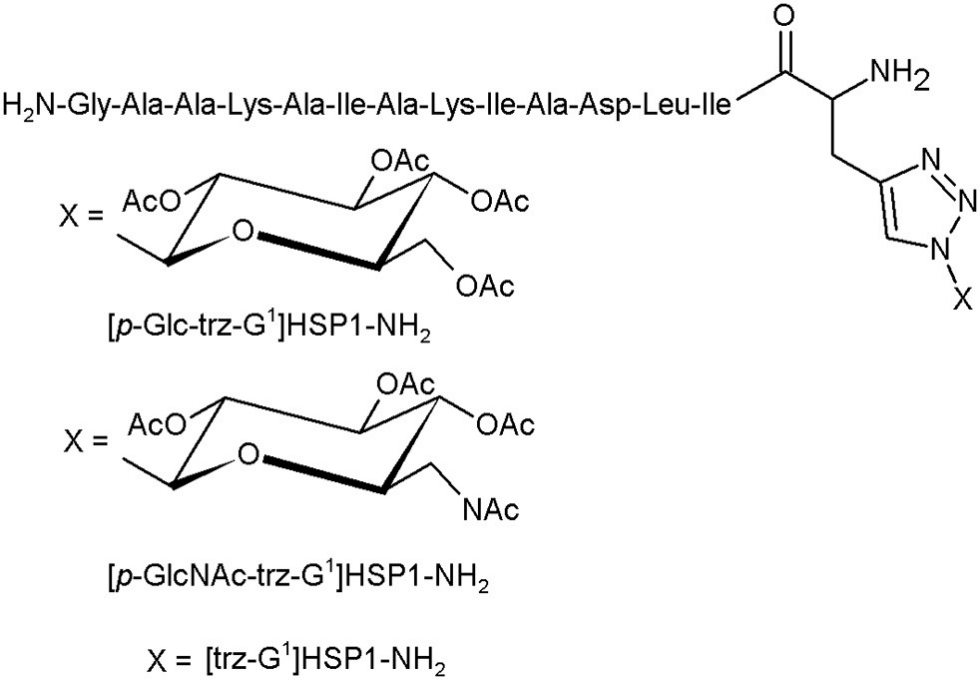
The glycotriazole-peptide derivatives (Junior et al., 2017).

The glycotriazole-peptide derivatives, proposed by Junior et al., consist of the 1,2,3-triazolyl and monosaccharide moieties attached to amino acid residues (Figure 5). They proposed the glycotriazole-peptide from hylaseptin-P1 (HSP1), an antimicrobial peptide characterised by a 14-amino acid sequence C-terminal amide (HSP1-NH_2_). In this context, the peptide chain was linked by the stable triazolyl bridge to the saccharide moiety (Junior et al., 2017).

The antibacterial properties of HSP1-NH_2_, [*p*-Glc-trz-G^1^]HSP1-NH_2_, [*p*-GlcNAc-trz-G^1^]HSP1-NH_2_, and [trz-G^1^]HSP1-NH_2_ were tested against gramme positive (*S. aureus, S. agalactiae*) and gramme negative strains (*P. aeruginosa, E. coli*) and compared with chloramphenicol. Except the *S. agalactiae*, almost all structures presented an antibacterial activity against the investigated species. Noteworthily, these assays indicated that the glycosylation promotes slight antibacterial activity. However, the glycotriazole-peptides and the [trz-G^1^]HSP1-NH_2_ displayed enhanced antifungal abilities compared to HSP1-NH_2_. The peptide HSP1-NH_2_ has no activity against *Candida* spp. strains. The triazole derivatives presented visibly higher antifungal activity, suggesting the importance of the triazole moiety in this process (Junior et al., 2017).

#### Structures Containing the Triazole Motifs and Used as Treatments in HIV

Lack of vaccines against human immunodeficiency virus type 1 (HIV-1) is a serious problem for public health. There is an urgent need to develop novel antiretroviral agents for HIV-1 treatment. Currently inhibitors applied in antiretroviral therapies target the viral enzymes: protease, integrase, and transcriptase (Rosemary Bastian et al., 2015). The virus attacks T-cells and macrophages by fusion of the viral membrane with target cell membrane. In this context, the entry of the virus into host cells is a good drug target. In this process, an important role is displayed by the glycoprotein from viral envelope, derived from the proteolytic cleavage of a gp160 precursor into the gp120 surface protein and the gp41 transmembrane protein. McFadden et al. proposed the peptide triazole derivative of 12p1, HNG-156, including a ferrocenyl triazole-substituted conjugate and binding to gp120 with an equilibrium dissociation constant of 7 nM KD, in contrast to the 2,600 nM KD value for 12p1. The study demonstrated that the HNG-156 is non-cytotoxic and has anti-HIV type 1 activity. The triazole peptide reacts well with all tested inhibitors (McFadden et al., 2012). Rashad et al. examined the peptide triazoles that inhibit HIV-1 cell infection and suppress gp120 receptor binding. The peptide triazole derivatives were stable to chymotrypsin and trypsin (Rashad et al., 2015).

### Triazole-Modified Peptidomimetics With Anticancer Properties

**Figure 6:**
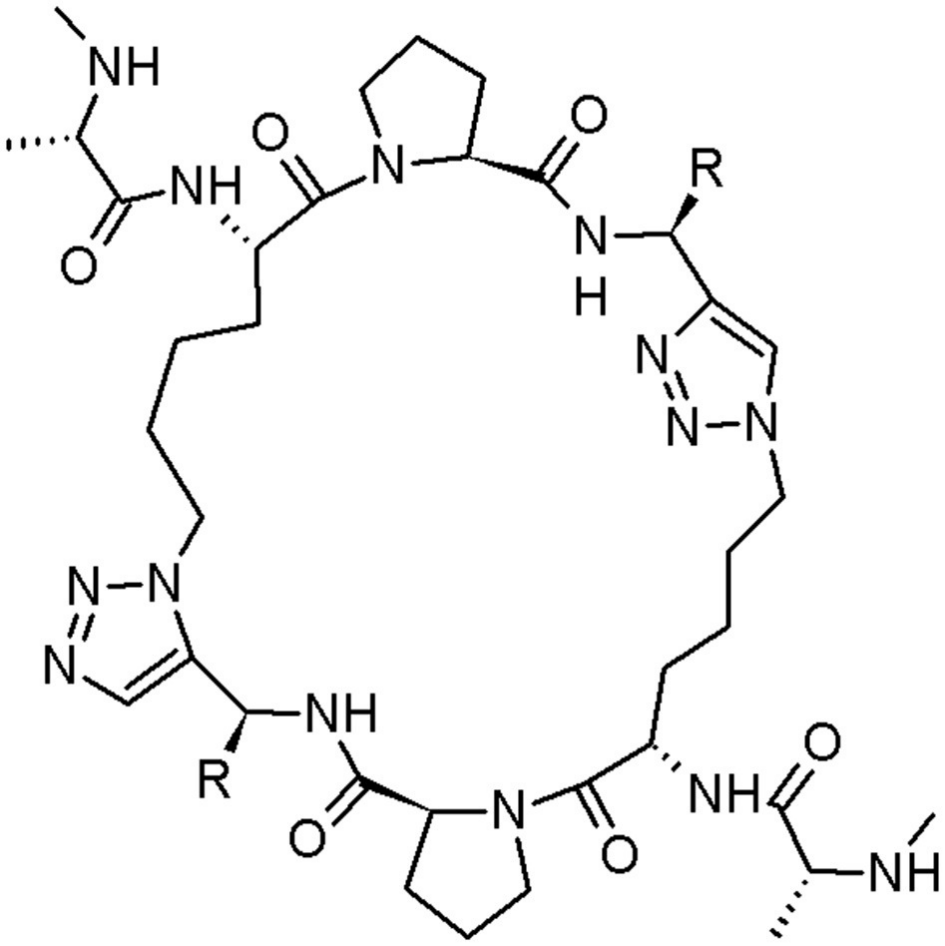
The cyclic peptidomimetic of Smac (structure **2**: R = phenyl; structure **3**: R = benzyl) (Sun et al., 2010).

Cancer is one of the most dreaded disease, characterised by metastasis, invasion, and uncontrolled cell growth (Megally Abdo and Kamel, 2015). Chemotherapy, radiation, and surgery are the major techniques among many, contemporarily applied for cancer treatment. Chemotherapy, the most used method (Shi et al., 2013), is regularly chosen in cancer cases where it has metastasised in the organism. The discovery of innovative anticancer drugs, presenting selectivity, and diminished side effects for regular cells, is a challenge (Ajmal et al., 2019). Sun et al. designed two cyclopeptide mimetics of second mitochondria-derived activator of caspase (Smac) with replacement of two amide bonds with two triazole moieties (Sun et al., 2010; Figure 6).

Two above-mentioned peptidomimetics bind to cIAP-1, cIAP-2 (inhibitor of cancer necrosis), and XIAP (blocking apoptotic pathways by binding with low nanomolar affinities and inhibiting caspases activity). Moreover, they can reclaim the activity of caspase-3/-7 and caspase-9, inhibited by XIAP. The structure **2** is ~5-8 time more efficient inhibitor of cell growth in two cell lines than compound **1** (Sun et al., 2010). For more caspase inhibitors, please see the dedicated chapter in this review.

According to the literature surveys, substituted 1,2,4-triazole, such as Anastrozole, Letrozole, and Vorozole are significant chemotherapeutics used in the treatment of breast cancer (Megally Abdo and Kamel, 2015). Baharloui et al. have pointed that the two peptides GLTSK and GEGSGA, including the triazolyl moieties, displayed significant anticancer properties against breast and colon cancer cells. Moreover, the peptidomimetics containing a triazole moiety presented higher cytotoxic activity on MDA-MB-231 cells rather than on MCF-7 cells (Baharloui et al., 2019).

**Figure 7:**
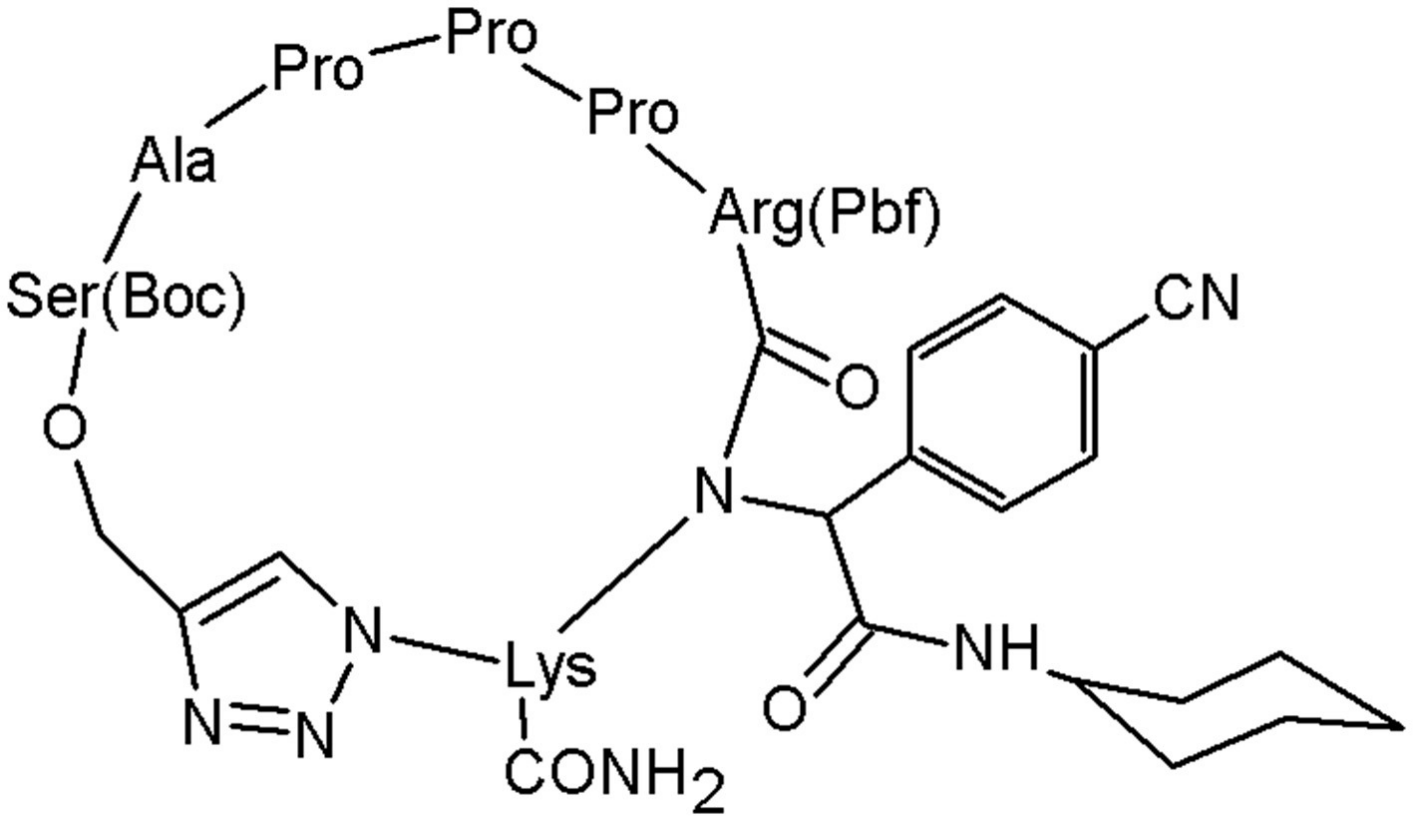
The peptidomimetic active against lung cancer cells, including a triazole moiety (Tahoori et al., 2014).

Tahoori et al. designed a molecule, which is highly active against lung cancer cells (Tahoori et al., 2014; Figure 7).

The structure has revealed the prospective to be used in tumour treatments by inducing apoptosis in some cancer cells known to have transformed ras oncogene. The anticancer activity was examined *in vitro* and this peptidomimetic has shown a significant activity against cancer cells with mutated ras oncogene such as A549, C26, and PC3 cells. This study demonstrates the role of the 1,2,3-triazole motif in the peptides bioactivity (Tahoori et al., 2014).

### Triazolyl-Containing Peptidomimetics as Human Enzymes Inhibitors

Enzymes play a pivotal role in all living organisms. Their dysfunctions and hyperactivity may lead to either disorders and lethal diseases (Copeland, 2005). Drug design based on enzyme inhibition mechanism is known for years and now it is a broadly investigated area, including low-molecular bioactive compounds (Hałdys et al., 2021), through macrocycles (Mallinson and Collins, 2012), and peptides or peptidomimetics on a final note (Gudapaty et al., 1985). Unfortunately, peptides are highly susceptible to enzymatic degradation and due to this obstacle, various modifications must be applied in order to increase the structure stability *in vivo* (Errante et al., 2021; Ledwoń et al., 2021). One of the most interesting approaches to solve this problem is the triazole moiety incorporation, leading to the peptidomimetic sequences with specific structural assets and enhanced life-time in cells (Fabbrizzi et al., 2014). In this review, we report a spectrum of scientific papers concerning peptidomimetics including the triazole ring, presenting significant inhibiting properties frequently supported by enzymatic selectivity.

#### Arginine Deaminase-4

The protein arginine deaminase-4 (PAD-4) belongs to the protein arginine deaminases (PAD) family, calcium-dependent enzymes highly involved in cellular growth and differentiation. Overactive PAD-4 leads to augmented citrullination process, correlated with several pathologies, such as multiple sclerosis (MS), Alzheimer's disease, and rheumatoid arthritis. Therefore, there is a remarkable requirement of chemicals able to decrease the PAD-4 activity. Trabocchi et al. (2015) synthesised a library of peptidomimetic compounds with an N*-* or C-terminal guanidino group. All sequences were obtained via copper(I)-catalysed 1,3-Huisgen cycloaddition and it resulted with 16 different 1,2,3-triazole derivatives. In Table 5 we reported the data of *in vitro* inhibition assays performed with two different concentrations of each compound, and then compared with chloroamidine (irreversible and commercially available inhibitor of PAD-4). Five molecules were inactive, while the rest achieved 5-41% or 16-99% inhibition at 1 and 10 mM concentration, respectively. Besides, the chloroamidine showed inhibitory effect of 24% (C_M_ = 1 mM) and 36% (C_M_ = 10 mM). Molecular modelling calculations have demonstrated that the bond formation between the 1,2,3-triazole ring and Arg374 or Trp347 residues is particularly favoured, and this preference depends on the lengths of the side chains (Trabocchi et al., 2015).

**Table 5:** The most effective PAD-4 inhibitors: commercially available chloroamidine and three 1,2,3-triazole-modified peptidomimetics **8**, **14**, and **16** (Trabocchi et al., 2015).

**PAD-4 inhibitor**	**Inhibitory effect on PAD-4**	**Chemical structure**
**Chloroamidine**	24% for C_M_ = 1 mM 36% for C_M_ = 10 mM	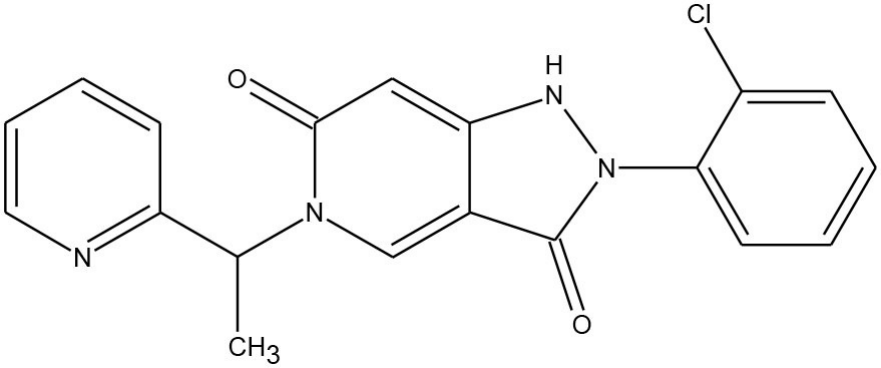
**(8)**	39% for C_M_ = 1 mM 99% for C_M_ = 10 mM	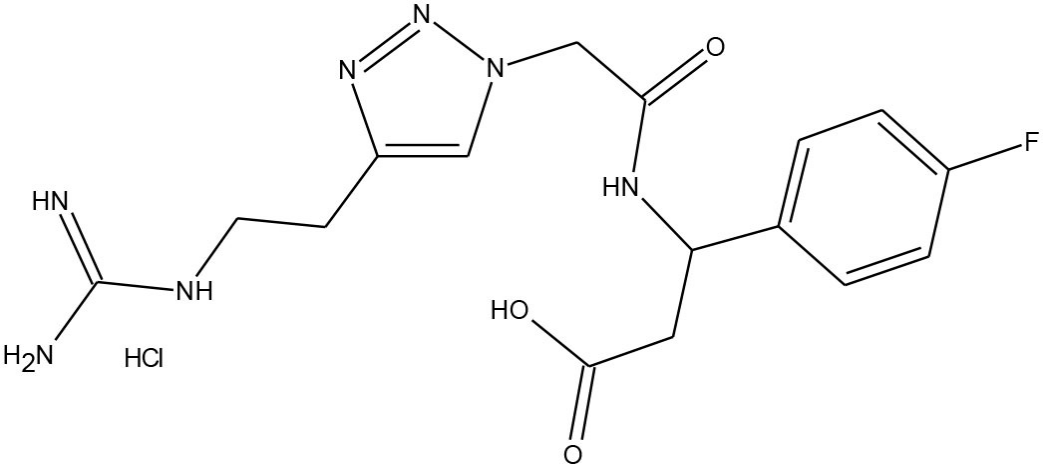
**(14)**	41% for C_M_ = 1 mM 99% for C_M_ = 10 mM	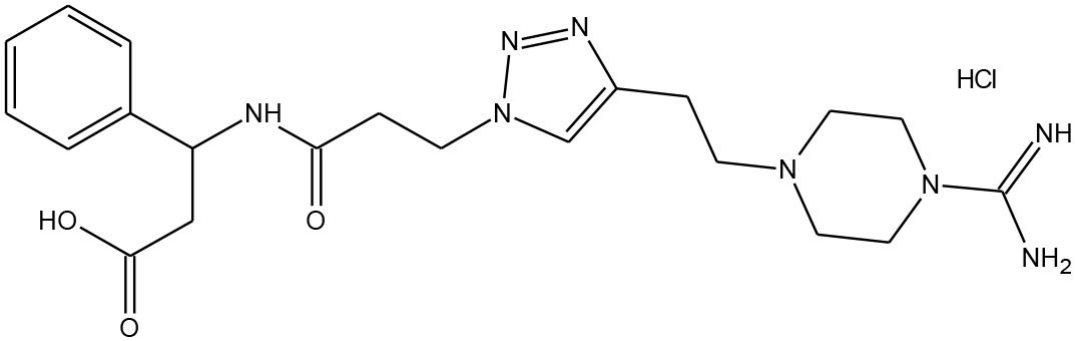
**(16)**	18% for C_M_ = 1 mM 99% for C_M_ = 10 mM	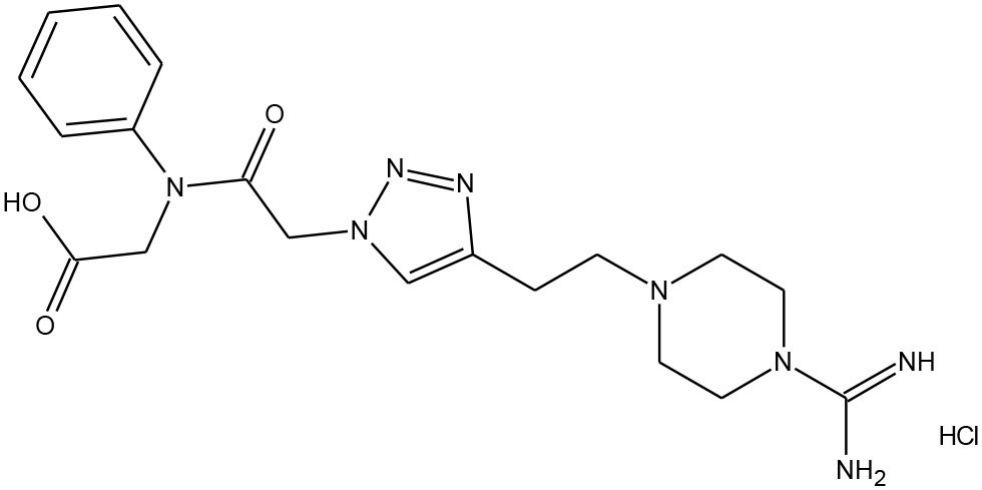

#### Calpain-2

Calpains, a family of 15 neutral proteases, influence the wide range of cellular functions. Their calcium-dependent mechanism of action is not consistent for each one of the fifteen family members, as some of them require lower or higher calcium concentrations (Potz et al., 2016; Baudry, 2019). Increased release of Ca^2+^ may result in calpain overactivation, then it promotes cellular apoptosis and results in organ dysfunctions, e.g., brain, eyes, heart, lungs, pancreas, kidneys, vascular system, and skeletal muscles (Potz et al., 2016). As a consequence, calpains inhibitors are contemporary investigated and among the recently described molecules, a few contain the triazole ring.

In 2012, Pehere and Abell proposed an optimised procedure for the macrocyclic peptidomimetics synthesis. Additionally, they studied the impact of β-strand geometry on the calpain-2 inhibition efficiency (Pehere and Abell, 2012). It is proved that linear peptides constrained into a well-organised conformation, e.g., β-strand, can offer significant advantages relevant for their bioactivity. In this framework, the Huisgen cycloaddition is a very promising platform useful for the synthesis of macrocycles, successfully mimicking the β-strand geometry. These modifications improve peptide biostability and limit the conformational mobility, that is exceptionally important in the context of inhibitors design. Interestingly, the conformationally preferred C-terminal aldehyde group, together with the amino acid hydrophobicity were noticed and figured during the synthesis.

A fluorescence-based assay was used to determine *in vitro* activity of the obtained products. IC_50_ values of remarkably potent molecules were calculated as 97 and 89 nM for two 21-membered macrocyclic aldehydes. This was then explained by the β-strand conformation adopted by these macrocycles. Notably, two non-cyclic aldehydes also investigated in this research were much less potent inhibitors than their cyclic analogues. Thereby, the importance of the depicted structural modifications was demonstrated.

#### Ghrelin *O*-acyltransferase

Ghrelin, a 28-residue acylated peptide with an n-octanoyl (C8) group on Ser3, is primarily generated by the stomach (Kojima et al., 1999). It is particularly implicated in processes associated with nutrition, being able to stimulate appetite, participating in insulin secretion and glucose regulation (Zhao et al., 2015). However, ghrelin was found as an important factor in other metabolic functions (Moulin et al., 2013). Ghrelin *O*-acyltransferase (GOAT), identified in 2008, catalyses the acylation of both proghrelin and the mature ghrelin (Yang et al., 2008). The unique nature of this process creates an ideal target for ghrelin activity inhibitors design.

Houghland and his group have designed 10 different, short peptidomimetics bearing a 1,2,3-triazole-linked lipid (Zhao et al., 2015). The previously mentioned hydrophobicity of residues was respected also in this case. The performed research has proven that the exchange of easily hydrolysed ester or amide linkages with the biostable triazole linkage is tolerated by the human GOAT. Additionally, the attachment of hydrophobic groups gains the potency and provides visibly better results in the context of GOAT inhibition (IC_50_ value of 0.7 μM).

#### Cysteine Proteases

The family of cysteine proteases is a well-described and known for years group of enzymes, able to degrade proteins (Chapman et al., 1997). However, their role in human biology cannot be considered only in this category. Cysteine proteases can also regulate apoptosis, immune responses, and prohormone managing, with addition to the processing of extracellular matrix (ECM) remodelling pathway (Chapman et al., 1997). Thereupon, it has been assumed that cysteine proteases can be partially responsible for collagen degradation in cells. Two enzymes belonging to the above-mentioned group, caspases, and cathepsins, have been described in this chapter, as their inhibitors containing the triazole moiety were recently defined and examined.

##### Caspases

An excellent and detailed review on different classes of caspases was published in 2015 by Poreba et al. (2015), reporting their properties, substrate specificity, and therapeutic strategies. Mainly, caspases are involved in numerous biological cell mechanisms, such as apoptosis, inflammation, differentiation, and survival in general. The dysregulation of caspases leads to many pathological states, e.g., connected to cardiovascular and nervous systems or carcinogenesis. For example, caspase over expression is supposed to be responsible for the non-regulated proliferation and tumour cells migration, thus resulting in severe illnesses or patients death (Gora and Latajka, 2015).

Disrupted apoptotic pathways occurring in different cancer types can be explained, among others, by the increased amount of inhibitors of apoptosis proteins (IAPs), which deactivate proteolytic caspases (Le Quement et al., 2011). The second mitochondria-derived activator of caspase, Smac protein, is an inhibitor of one of the IAPs classes. In 2011, Nielsen and co-workers described the solid-phase synthesis of a Smac-derived library of tetrapeptides, based on the AVPF sequence. Including, Smac peptidomimetics with triazole-prolines and biarylalanine motifs in the structure. The biological screening of the synthesised molecules, including the diversification between *cis* and *trans* isomers, have shown promising bioactive properties. For example, in the case of triazole-proline containing Smac mimetics, their *cis* analogues were more potent than *trans*. Triazoloproline and biarylalanine libraries revealed IAP inhibition, therefore proving the relevance of the structural modifications.

Although this review is essentially focused on peptidomimetics, relevant papers regarding triazole-containing small molecules are described below. In 2015, Guo et al. investigated three series of 1,4-disubstituted 1,2,3-triazoles as inhibitors of caspase-3 and caspase-7 (Guo et al., 2015). Among the most potent caspase-3 and caspase-7 inhibitors with IC_50_ values in nM range, it was found that the crucial role for their activity was played by the N-terminal incorporation of the urea group. Molecular docking studies have confirmed the formation of hydrogen bonds in the binding site of caspase-3, relevant for the inhibition mechanism.

Isosteric replacement of an amide bond by the 1,2,3-triazole moiety was in the focus on another work by Corredor et al. (2015). All the synthesised compounds were examined as inhibitors of apopotosome-dependent activation of procaspase-9. It was noted that the unexpected formation of β-lactams occurs during the synthesis. Also, these analogues were chosen to be tested with procaspase-9. The obtained IC_50_ values in the nM range were satisfying enough. Moreover, for the isomeric molecules, the β-lactam scaffold has shown better results.

Another detailed study on the caspases 1-3 and 6-9 was performed by Leyva et al. (2010). The substrate activity screening method (SAS) was applied to identify the substrates of caspase-6 and caspase-9. Then, the reporter groups in the most potent substrates were replaced with the pharmacophore including the 1,2,3-triazole moiety. The study led to three non-peptide inhibitors with irreversible character, showing high deactivating properties of caspases and weak or no inhibition of other cysteine proteases.

##### Cathepsins

In 2012, Valverde et al. presented an interesting study reporting for the first time the use of CuAAC reaction “for the assembly of unprotected peptide fragments into a bioactive triazole-containing protein” (Valverde et al., 2012). They indicated the importance of the triazole moiety in forming a secondary structure by hydrogen bonding and other stabilising interactions.

In the above-mentioned paper, the authors use cystatin A as a potent inhibitor of cysteine proteases, including cathepsins. It complies with requirements of a molecule well-suited into the active site cleft, e.g., the N-terminal glycyl residue, the C-terminal β-hairpin loop and, placed centrally, the β-hairpin loop with the QXVXG motif. Therefore, the 97-amino acids sequence of cystatin A has been divided into three shorter fragments. Their SPPS was performed and the triazole linker was inserted to connect them into one chain. The inhibition screening for cathepsin B, H, and L was performed and inhibitory results in line with these presented in the literature were observed (*K*_i_ values in nanomoles). The synthesised molecules adopt a 3D structure, comparable to the native proteins. In addition, the triazole-containing analogue displayed a proper interactions with the protein, thereby indicating its role in the biological mechanism.

A follow-up to Pehere and Abell work (Pehere and Abell, 2012), presented in the chapter on calpain II inhibitors, is the screening of 11 novel macrocyclic compounds against a panel of proteases, including cathepsins and calpain II (Pehere et al., 2013), compared to four acyclic analogues. All of them consisted of C-terminal aldehyde, favouring the interaction with the active cleft of the targeted protease. All peptidomimetics were efficiently inhibiting cathepsin S, with IC_50_ values <5 nM. Eight out of the fifteen tested compounds were also visibly active against cathepsin L (IC_50_ between 10 and 50). Effect of macrocyclization is supposed to enhance the potency of inhibition, due to the lower IC_50_ values found for the cyclic mimetics.

A recently published article delineating triazole-containing peptides and azapeptides as potential inhibitors of cathepsins K and S is also worth to be noticed (Galibert et al., 2018). Lalmanach and his group have synthesised four compounds, among which the ones containing the 1,4-disubstituted 1,2,3-triazole moiety or a semicarbazide bond were reported, thus replacing the C_α_ of glycine by a nitrogen atom. The screening resulted with noticeably better outcomes for aza-Gly analogues respect to triazole-mimetics. However, both groups have shown an interesting activity (*K*_i_ around nM vs. mM range, respectively). Indeed, molecular modelling studies explain the preference of the semicarbazide bond, as found for the foremost analogues.

#### Serine Proteases

According to the detailed and undoubtedly valuable review published in 2000 describing serine protease family, this group of enzymes covers one-third of all classified proteases (Hedstrom, 2002). Four subclasses, that are chymotrypsin, subtilisin, carboxypeptidase Y, and Clp protease with the classic catalytic triad (His, Asp, and Ser) can be distinguished (Rawlings, 2000). Afterwards, some additional subclasses have been found (for more details, see the cited review). Overactive serine proteases may lead to interrupted gastrointestinal physiology, as they are secreted in the pancreas in order to be activated for digestive purposes. Thus, this pathological state can be found in Crohn's disease or Ulcerative Colitis patients (Denadai-Souza et al., 2018).

A different trypsin-like serine protease, the fibrinolytic enzyme plasmin, takes part in the blood fibrin clots degradation (Saupe and Steinmetzer, 2012).

In this chapter, we mention some new peptidomimetic inhibitors of serine proteases, possessing the triazole bridge, present in the structure.

##### Trypsin-Like Serine Proteases

There are a few relevant triazolyl-containing peptidomimetics, that can be classified as trypsin-like serine proteases inhibitors. Most of them have been designed on the grounds of the sunflower trypsin inhibitor-1 (SFTI-1). Herein, we report several forms that have been reported to decrease the enzyme activity in a relatively efficacious way. In 2013, Fittler et al. designed, synthesised, and tested a library consisting of 22 compounds, in order to enhance the inhibitory activity of the parent STFI-1 structure. Among these modifications, 16 compounds were represented as the triazole-derivatives (Fittler et al., 2013). Apart the triazolyl bridge incorporation, other refinements were applied such as sequence shortening and non-natural amino acids coupling. Nanomolar activity of the synthesised molecules was then explained by *in silico* experiments, thus indicating an additional proton donor-acceptor interaction of basic ε^2^-N in histidine and acidic residues in the protease active cleft.

Similar work focused on the introduction of 1,4-disubstituted 1,2,3-triazoles into the peptide sequences that were prepared by Empting et al. (2011). They proposed the disulphide-bridge replacement, with the idea in mind of conformation rigidity enhancement leading to a more effective activity than the STFI-1. The four analogues developed were then tested biologically. One of the 1,5-disubstituted 1,2,3-triazole peptidomimetics displayed nanomolar inhibitory power, in line with the results of disulphide-bridged STFI-1.

##### Plasmin

Saupe and Steinmetzer described the synthetic strategy to obtain potent and selective peptide inhibitors of plasmin (Saupe and Steinmetzer, 2012). Plasmin, already mentioned in the introduction of this section, is a non-specific trypsin-like serine protease, participating in blood fibrin clots degradation. Hence, inhibitors of plasmin can be employed in the hyperfibrinolysis treatment, occurring for instance, in organ transplantation.

The copper-catalysed azide alkyne cycloaddition and solid-phase peptide synthesis yielded 12 different peptidomimetics, including rigid triazole rings in the side chains. All analogues were examined against five different proteases, including plasmin. The lowest and particularly interesting value obtained for one of the structures, that is *K*_i_ = 0.77 nM, indicated the effectiveness in the inhibitory activity and the selectivity among other proteases (thrombin, factor Xa, and aPC).

##### Dipeptidyl Peptidase IV

There is a wealth of patents filed in the area of dipeptidyl peptidase IV (DPP IV) inhibitors. Major part of the structures includes structural modifications to achieve higher stability and selectivity. A highly applicative article reporting patents in the field of DPP IV inhibitors featuring peptides, peptidomimetics, and triazolyl moieties, was presented in 2011 by Mendieta et al. and certainly is worth to be mentioned in the present review (Mendieta et al., 2011).

Enzymes and corresponding peptidomimetic inhibitors with triazole-modified sequences described in this review, are summarised in the Table 6.

**Table 6:** Enzymes and corresponding peptidomimetic inhibitors with triazole-modified sequences, described in this review.

**Enzyme class**	**Enzyme name**	**References**
Arginine deiminases	Arginine deiminase-4	Trabocchi et al., 2015
Neutral, Ca^2+^-dependent proteases	Calpain-2	Pehere and Abell, 2012
Acyltransferases	Ghrelin *O-*acyltransferase	Zhao et al., 2015
Cysteine proteases	Caspases	Le Quement et al., 2011 *Non-peptide triazole-derivatives:* Guo et al., 2015 Corredor et al., 2015 Leyva et al., 2010
	Cathepsins	Valverde et al., 2012 Pehere et al., 2013 Galibert et al., 2018
Serine proteases	Matriptase	Fittler et al., 2013 Empting et al., 2011
	Plasmin	Saupe and Steinmetzer, 2012
	Dipeptidyl peptidase IV	*Review on patents on DPP IV inhibitors:* Mendieta et al., 2011

## Conclusions

In this review we have highlighted the most relevant studies concerning the synthesis of peptidomimetics containing the 1,2,3-triazolyl moiety and the corresponding biological activity. Peptides containing triazolyl rings are a typical example of peptidomimetics. They demonstrate all the advantages over classic peptides. Both efficient synthetic methods and biological activity make these systems interesting and promising object of research.

In particular, triazolyl-containing peptidomimetics can be widely applied as anticancer, antibacterial, antifungal, and antiviral agents. It is worth to notice that these modifications provide higher bioactivity and stability compared to the non-modified analogues. Our insights may be an interesting summary for further investigations in drug design including triazolyl moieties.

## Author Contributions

AS, PL, PR, AMP, and RL collected the articles mentioned in the review and participated in manuscript preparation. All authors contributed to the article and approved the submitted version.

## Conflict of Interest

The authors declare that the research was conducted in the absence of any commercial or financial relationships that could be construed as a potential conflict of interest.
